# Testing of the WHO hand hygiene self-assessment framework

**DOI:** 10.1186/1753-6561-5-S6-P130

**Published:** 2011-06-29

**Authors:** AJ Stewardson, B Allegranzi, T Perneger, H Attar, D Pittet

**Affiliations:** 1 University of Geneca Hospitals, Geneva, Switzerland; 2 WHO, Geneva, Switzerland

## Introduction / objectives

The Hand Hygiene Self-Assessment Framework (HHSF) was conceived by the WHO 1^st^ Global Patient Safety Challenge as a systematic self-assessment tool to provide a situation analysis of hand hygiene (HH) resources, promotion and practices within healthcare facilities.

## Methods

Development consisted of three phases: initial drafting, usability testing and reliability testing. The HHSF draft was developed by a team of experts, based on the key elements of the WHO multimodal HH improvement strategy. For usability testing, 42 HC facilities were invited to score their facility and complete a feedback survey. For inter-rater reliability testing, two users in each facility independently completed the HHSF. The reliability of each indicator, component sub-total and the overall score was estimated by using the variance components model. After each phase, the tool was examined with regard to the need for modification.

## Results

27 indicators were selected during drafting. 26 facilities in 19 countries responded (62% response rate) for usability testing. Results reflected a broad range of HH promotion and practice with total scores ranging from 35–480 (mean, 262). The HHSF took <2 hours to complete for 21 facilities. The majority agreed that the HHSF was “easy to use” (23/26) and "useful for establishing facility status with regard to HH promotion" (24/26). Complete reliability responses were received from 41 facilities in 16 countries. The reliability for the total score for the HHSF and the subtotal of each of the five components ranged from 0.54 to 0.86. Seven of the 27 indicators had poor reliability; these were examined for potential flaws and modified accordingly.

## Conclusion

Results support the usability and reliability of this tool in the promotion of HH in healthcare.

## Disclosure of interest

None declared.

